# Possible link between dental diseases and arteriosclerosis in patients on hemodialysis

**DOI:** 10.1371/journal.pone.0225038

**Published:** 2019-12-13

**Authors:** Taro Misaki, Akiko Fukunaga, Yoshitaka Shimizu, Akira Ishikawa, Kazuhiko Nakano

**Affiliations:** 1 Division of Nephrology, Seirei Hamamatsu General Hospital, Hamamatsu, Shizuoka, Japan; 2 Department of Nursing, Faculty of Nursing, Seirei Christopher University, Hamamatsu, Shizuoka, Japan; 3 Division of Dentistry, Seirei Hamamatsu General Hospital, Hamamatsu, Shizuoka, Japan; 4 Ai Dental Clinic, Hamamatsu, Shizuoka, Japan; 5 Department of Pediatric Dentistry, Division of Oral Infection and Disease Control, Osaka University Graduate School of Dentistry, Suita, Osaka, Japan; Istanbul Universitesi Dis Hekimligi Fakultesi, TURKEY

## Abstract

**Background:**

Patients on hemodialysis must undergo this procedure at a hospital three times weekly and might be unable to visit a dentist. In addition, dentists might hesitate to provide oral care because such patients tend to bleed because they are medicated with anticoagulants, are susceptible to bacterial infections, and might have unusual drug reactions. We postulated that patients on hemodialysis have worse oral status than healthy people, which in turn might predispose such patients to systemic complications.

**Methods:**

We compared the status of dental caries and periodontal diseases among 80 patients on hemodialysis and 76 healthy individuals (controls) using the decayed, missing, or filled teeth (DMFT) index, total number of C4 teeth (destruction of the entire tooth crown), and periodontal pocket depth. Clinical data were analyzed after all patients on hemodialysis and controls provided written, informed consent to participate in the study.

**Results:**

Total number of C4 teeth (*p* = 0.021), missing teeth (MT) index (*p* = 0.0302), and DMFT index score ≥ 24 (*p* = 0.017) were significantly higher in patients on hemodialysis than controls. Pulse pressure (*p* = 0.0042) and the prevalence of a history of heart disease such as angina pectoris and acute myocardial infarction (*p* = 0.029) were higher in patients on hemodialysis with higher (≥ 24) than lower (< 24) DMFT index scores. Periodontal pocket depth was not significantly different between these two groups.

**Conclusion:**

Worse status of dental caries is possibly associated with arteriosclerosis among patients on hemodialysis.

## Introduction

Patients with IgA nephropathy have greater dental caries than healthy individuals, and dental caries status is associated with proteinuria in these patients [1]. This suggests that worse dental caries might be associated with end-stage renal failure, although various diseases can certainly lead to renal failure [2]. Moreover, patients on hemodialysis (HD) must undergo this procedure at a hospital three times a week and might be unable to visit a dentist. In addition, dentists might hesitate to provide oral care to such patients who tend to bleed because they are medicated with anticoagulants, are susceptible to bacterial infections, and might have unusual drug reactions. An association between oral diseases and systemic health has been identified [3, 4], and patients on HD are more susceptible to dental caries than healthy individuals [5]. Furthermore, an association between periodontitis and chronic kidney disease (CKD) has been identified [6–8]. It is reported that risks of all-cause and cardiovascular mortality of patients on HD are associated with moderate to severe periodontitis [7]. In addition, arteriosclerosis is considered to progress in patients on HD via CKD with mineral and bone disorders [9] and cardiovascular diseases comprise the most common cause of death among patients with CKD [10]. In this study, a cross-sectional analysis of data derived from patients on HD and age-matched healthy individuals was conducted. The aims of the study were to compare the oral condition of patients on HD and healthy individuals and extract factors that affect the health of patients on HD by oral condition. We postulated that the oral status of patients on HD is worse than that of healthy people and that this status might be associated with systemic complications.

## Methods

### Participants and clinical data

We asked all 94 outpatients on HD at Seirei Hamamatsu General Hospital, Hamamatsu, Japan, to participate in this study and 80 patients gave their consent. We collected information from 80 outpatients between 2017 and 2018. Clinical data [age, sex, height, dry weight, body mass index (BMI), systolic blood pressure, diastolic blood pressure, pulse pressure, white blood cell (WBC) count, hemoglobin, platelets, serum albumin, serum creatinine, C-reactive protein, intact parathyroid hormone (intact PTH), calcium, phosphate, blood sugar, Glico albumin, diabetes mellitus, ejection fraction, history of heart disease (angina pectoris, acute myocardial infarction), cerebral infarction, and cerebral hemorrhage] were collected after patients provided written, informed consent to participate in this study. Age-matched healthy individuals (n = 76) who attended Ai Dental Clinic, Hamamatsu, Shizuoka, Japan, were included as controls. Healthy individuals were defined as those who were not on dialysis and were extracted from dental patients of a private dental clinic at Hamamatsu, Japan, according to the age and sex distribution of patients in the HD group.

### Evaluation of dental caries status and periodontal disease

Dental caries status among the 80 patients on HD and 76 controls was evaluated by two dentists using conventional methods at the Department of Dentistry, Seirei Hamamatsu General Hospital and Ai Dental Clinic. The numbers of total, decayed (DT), missing (MT), and filled (FT) teeth were clinically assessed. Then, the DMFT score was calculated using the total numbers of D, M, and F as previously described [1, 11]. The intensity of dental caries was evaluated by the lesion extending to the enamel (C1), dentin (C2), and pulp space (C3). In addition, the lesion showing destruction of the entire tooth crown was evaluated as C4. As for periodontal conditions, periodontal pocket depth was measured using the World Health Organization probe, and then the rates of those who showed ≥ 4 mm and ≥ 6 mm periodontal pocket depth were calculated.

### Statistical analysis

All results are expressed as means ± standard deviation (SD). When a significant difference was identified, values were further analyzed using Fisher’s protected least significant difference tests or chi-squared tests and multivariate logistic regression analysis models adjusted for age and sex. A two-tailed value of *p* < 0.05 was considered significant. Data were statistically analyzed using StatView software (SAS Institute Inc., Cary, NC, USA).

### Ethics approval

This study fully complied with the Declaration of Helsinki (64^th^ WMA General Assembly, Fortaleza, Brazil, 2013), and the Ethics Committee at Seirei Hamamatsu General Hospital approved the study protocols (approval no. 2382). The protocols were explained in detail to all participants, who then provided written, informed consent to participate in the study.

## Results

### Dental status of patients on HD compared with controls

Table 1 summarizes the dental status of 80 patients on HD and 76 controls. Age and sex distributions did not significantly differ between groups at the time of presentation. Values for DT, FT index, and DMFT index and the numbers of subjects who showed ≥ 4 mm periodontal pocket depth, ≥ 6 mm periodontal pocket depth, C1, C2, and C3 did not significantly differ between the two groups (Table 1). However, patients on HD had a significantly higher prevalence of C4 (*p* = 0.021), a higher MT index (*p* = 0.0302), and higher DMFT index scores (≥ 24; *p* = 0.017) than healthy controls.

**Table 1:** Characteristics of patients on hemodialysis and controls.

Characteristics	Control (n = 76)	HD patients (n = 80)	*P*-value
Age (y; mean ± SD)	66.6 ± 12.1	67.3 ± 12.2	0.7305
Sex (M/F)	44/32	48/32	0.7909
DT index score	1.6 ± 2.2	1.9 ± 2.9	0.4975
**Total number of C4 teeth**	**0.2 ± 0.7**	**0.7 ± 1.5**	**0.0210**
**MT index score**	**5.2 ± 7.4**	**8.0 ± 8.7**	**0.0302**
FT index score	10.6 ± 5.5	9.1 ± 6.5	0.1288
DMFT index score	17.3 ± 6.7	19.0 ± 6.6	0.1288
**DMFT index score** ≥ **24 (%)**	**13.2**	**28.7**	**0.0170**
Periodontal pocket > 4 mm (%)	84.7	86.5	0.7633
Periodontal pocket > 6 mm (%)	30.6	23.0	0.3038

Bold values indicate statistical significance at *p* < 0.05.

### Clinical differences between high and low DMFT index scores among patients on HD

We analyzed 80 patients (mean age, 67.3 ± 12.2 years; male, n = 48; Table 2) according to DMFT index scores and classified them into high (≥ 24; n = 23) or low (< 24; n = 57) DMFT index groups. These two groups did not significantly differ in terms of sex, height, systolic blood pressure, WBC count, hemoglobin, platelets, serum albumin, serum creatinine, C-reactive protein, intact PTH, serum calcium, serum phosphate, blood sugar, Glico albumin, ejection fraction, diabetes mellitus rates, history of cerebral infarction, and cerebral hemorrhage. However, a high DMFT index score was significantly associated with more advanced age (*p* = 0.0018), lower dry weight (*p* = 0.006), lower BMI (*p* = 0.0077), lower diastolic blood pressure (*p* = 0.0395), higher pulse pressure (*p* = 0.0042), and a higher prevalence of a history of heart disease (e.g., angina pectoris and acute myocardial infarction; *p* = 0.029). Table 3 summarizes the results of the multivariate logistic regression analysis adjusted for age and sex. Pulse pressure > 80 mmHg and high DMFT index score (≥ 24) remained significantly associated (*p* = 0.0301), whereas more advanced age, lower dry weight (< 47kg) (*p* = 0.1645) (S1 Table), lower BMI (<20) (*p* = 0.4220) (S2 Table), lower diastolic blood pressure (<74 mmHg) (*p* = 0.0566) (S3 Table), and anamnesis of a history of heart disease (*p* = 0.1419) (S4 Table) did not remained significantly associated using multivariate logistic regression analysis adjusted for age and sex.

**Table 2:** Comparison between low and high DMFT index scores among patients on hemodialysis.

Characteristics	Low DMFT (< 24) n = 57	High DMFT (≥ 24) n = 23	*P-*value
**Age (y; mean ± SD)**	**64.6 ± 12.5**	**73.8 ± 8.4**	**0.0018**
Sex (M/F)	34/23	14/9	0.9209
Height (cm; mean ± SD)	161.2 ± 9.3	158.4 ± 10.5	0.2391
**Dry weight (kg; mean ± SD)**	**55.7 ± 12.2**	**47.7 ± 9.3**	**0.0060**
**BMI (kg/m**^**2**^**; mean ± SD)**	**21.3 ± 3.9**	**18.9 ± 2.5**	**0.0077**
Systolic blood pressure (mmHg; mean ± SD)	149.8 ± 23.3	157.15 ± 26.1	0.2237
**Diastolic blood pressure (mmHg; mean ± SD)**	**81.2 ± 12.9**	**74.0 ± 16.0**	**0.0395**
**Pulse pressure (mmHg; mean ± SD)**	**68.6 ± 19.9**	**83.0 ± 19.8**	**0.0042**
WBC (/ml; mean ± SD)	5609.8 ± 1521.4	5959.6 ± 2164.0	0.4148
Hemoglobin (g/dl; mean ± SD)	11.9 ± 1.1	11.9 ± 1.2	0.9575
Platelets (10^4^/ml; mean ± SD)	16.8 ± 5.2	19.4 ± 7.8	0.0822
Serum albumin (mg/dl; mean ± SD)	3.4 ± 0.4	3.4 ± 0.3	0.718
Serum creatinine (mg/dl; mean ± SD)	10.3 ± 3.1	9.7 ± 2.3	0.4137
C-reactive protein (mg/dl; mean ± SD)	0.3 ± 0.5	1.0 ± 2.7	0.0783
Intact PTH (pg/ml; mean ± SD)	157.1 ± 100.3	177.3 ± 117.7	0.4409
Serum calcium (mg/dl; mean ± SD)	8.6 ± 0.7	8.4 ± 0.7	0.2734
Serum phosphate (mg/dl; mean ± SD)	5.1 ± 1.2	5.1 ± 1.3	0.9755
Blood sugar (mg/dl; mean ± SD)	115.5 ± 39.0	110.5 ± 30.2	0.5848
Glico albumin (%; mean ± SD)	19.0 ± 2.6	17.1 ± 3.1	0.2173
Ejection fraction (%)	63.7 ± 11.2	62.5 ± 9.3	0.6528
Diabetes mellitus rate (%)	28.1	21.7	0.5660
**Anamnesis of heart disease rate (%)**	**10.5**	**30.4**	**0.0290**
Anamnesis of cerebral infarction rate (%)	14.0	8.7	0.5195
Anamnesis of cerebral hemorrhage rate (%)	5.3	8.7	0.5717

Bold values indicate statistical significance at *p* < 0.05. BMI: body mass index. DMFT scores: the numbers of total, decayed (DT), missing (MT), and filled (FT) teeth, intact PTH: intact parathyroid hormone, WBC: white blood cell.

**Table 3:** Association between pulse pressure > 80 mmHg and high DMFT index score in patients on hemodialysis.

Variables	Odds ratio (95% confidence interval)	*P*-value^a^
**Age**	**1.074 (1.019–1.133)**	**0.0080**
Sex (Male)	0.945 (0.336–2.662)	0.9149
**High DMFT** **(DMFT index score** ≥ **24)**	**3.444 (1.126–10.532)**	**0.0301**

Additive multivariate logistic regression models adjusted for age and sex were used for these analyses in patients on HD. The association between pulse pressure > 80 mmHg and high DMFT index scores (≥ 24) remained significantly different in a subsequent logistic regression analysis adjusted for age and sex. Independent variables were age, sex, and high DMFT index score (≥ 24). The dependent variable was pulse pressure > 80 mmHg. DMFT scores: the numbers of total, decayed (DT), missing (MT), and filled (FT) teeth.

^a^Bold values indicate statistical significance at *p* < 0.05.

## Discussion

To our knowledge, this is the first study to show the possibility that having worse dental caries is associated with high pulse pressure among patients on HD. Patients on HD had significantly worse dental caries and more missing teeth than healthy age-matched controls. The prevalence of a DMFT index score ≥ 24 was significantly higher among patients on HD than controls. These results indicate that patients on HD are not receiving sufficient oral care and treatment. The main reason might be that patients must undergo HD three times weekly at a hospital and might not have time to visit a dentist. Other reasons could be that patients on HD tend to bleed because they are medicated with anticoagulants, they are susceptible to bacterial infections, and they need to be cautious about drug reactions. Therefore, dentists might hesitate to provide dental treatment to such patients. Furthermore, patients with end-stage renal failure might originally have had a higher prevalence of dental caries. We previously reported that patients with IgA nephropathy have a higher frequency of dental caries than healthy individuals, and dental caries status is associated with proteinuria in such patients [1]. Patients on HD usually have reduced salivary flow or xerostomia because of HD, restricted oral fluid intake, side effects of drug therapy, and aging [5, 12]. Palmer et al. showed that poorer dental health among patients on HD is associated with early death, whereas preventive oral care is associated with prolonged survival [13]. Based on the above, we believe that patients on HD should be educated about the importance of regular dental checkups, and doctors with patients on HD should collaborate with dentists and exchange pertinent information. We initially thought that patients on HD might have more dental caries or periodontal disease than healthy controls. However, DMFT index score and periodontal pocket depth did not significantly differ between HD patients and controls (Table 1). Yue et al. reported a significantly higher DMFT index score among patients with CKD on HD than healthy controls (4.4 ± 3.9 vs 2.3 ± 2.5). However, they targeted a small group of patients (n = 30) with a mean age of 48.5 ± 12.7 years [5]. The mean age of our participants was 67.3 ± 12.2 years. Therefore, we considered that our healthy controls also had more caries due to aging and thus we could not determine a clear difference between patients on HD and controls. We evaluated periodontal disease using periodontal pocket depth, which may not have fully reflected the state of periodontal disease. More specific analyses using the parameters of periodontal attachment loss and prior history of confirmed periodontal disease are necessary in subsequent studies.

Our data were collected from a small outpatient cohort at our hospital and from healthy controls who attended a single dental clinic; thus, selection bias may exist because patients who visit dentists might have poorer oral status in general, and vice versa. The Ministry of Health, Labour and Welfare of Japan published DMFT data derived from 6,278 individuals in 2016; DMFT index scores were 17.1 and 19.2 for age groups 55–64 and 65–74 years, respectively [14]. The mean age of our participants was 66.6 years and mean DMFT score was 17.3 for controls. Thus, we believe that bias was not a factor in our selection of controls.

Patients on HD were checked for clinical data three times a week and laboratory data every month. We used these data from the day when participants initially agreed to participate in the study by providing informed consent. We used important laboratory parameters for patients on HD in this study. Patients on HD are malnourished [15], have renal anemia [16], are prone to infections [17], and many have diabetes [18]. Patients on HD have problems with calcium phosphate metabolism, subsequently causing arteriosclerosis. Therefore, many of them have complications of cerebral infarction and heart disease [9]. We found here that worse dental caries was associated with high pulse pressure among patients on HD, indicating that such patients might have arteriosclerosis. Reports indicate that arteriosclerosis progresses along with increases in pulse pressure difference and that this is associated with cardiovascular disease [19, 20]. Although the difference did not reach statistical significance in the logistic regression analysis, high DMFT score was significantly associated with a higher prevalence of a history of heart diseases such as angina pectoris and acute myocardial infarction (*p* = 0.029). However, the present study did not find sufficient evidence to determine the process through which dental caries contributes to the development of arteriosclerosis. *Streptococcus mutans* is a Gram-positive, oral streptococcal species that is a major pathogen of human dental caries [1]. It has been frequently detected in specimens of heart valves (69%) and atheromatous plaque (74%), suggesting that this microbe is a causative agent of cardiovascular disease [21]. In general, advanced dental caries results in the destruction of enamel and dentin on the tooth surface that exposes the pulp, which contains blood capillaries. Therefore, oral bacteria including *S*. *mutans* can enter the bloodstream at any time in daily life. We hope to study the pathogenesis of *S*. *mutans* in the future. Ruospo et al. also reported that risks of all-cause and cardiovascular mortality of patients on HD were associated with moderate to severe periodontitis [7]. Further studies are required to elucidate the involved mechanisms.

The present study has some limitations. First, we only showed that worse dental caries was associated with high pulse pressure among patients on HD. Whether persistent dental caries can induce arteriosclerosis remains to be confirmed in animal models. Second, we were unable to evaluate potential confounders such as diet and socioeconomic status. Finally, this study included a relatively small number of patients on HD from a single institution. Our results must be confirmed by future prospective and larger multi-institutional studies.

## Conclusion

Worse status of dental caries is possibly associated with arteriosclerosis among patients on hemodialysis.

## Supporting information

S1 Table Association between dry weight < 47kg and high DMFT index score in patients on hemodialysis. (DOCX)

S2 Table Association between BMI<20 and high DMFT index score in patients on hemodialysis. (DOCX)

S3 Table Association between diastolic blood pressure <74 mmHg and high DMFT index score in patients on hemodialysis. (DOCX)

S4 Table Association between anamnesis of a history of heart disease and high DMFT index score in patients on hemodialysis. (DOCX)
