# Supplementary material for: Possible link between dental diseases and arteriosclerosis in patients on hemodialysis
Source: PLoS One. 2019 Dec 13;14(12):e0225038. doi: 10.1371/journal.pone.0225038 (PMC6910673; doi:10.1371/journal.pone.0225038)
Supplement: S1 Table — (DOCX) [file pone.0225038.s001.docx]

**S1 Table: Association between Dry Weight < 47kg and High DMFT Index Score in Patients on Hemodialysis.**

| **Variables** | **Odds ratio (95% confidence interval)** | ***P*-value^a^** |
| --- | --- | --- |
| **Age** | **1.076 (1.013–1.143)** | **0.0180** |
| **Sex (Male)** | **0.068 (0.019–0.246)** | **0.0010** |
| High DMFT  (DMFT index score ≥ 24) | 2.546 (0.682–9.509) | 0.1645 |

Additive multivariate logistic regression models adjusted for age and sex were used for these analyses in patients on HD. The association between dry weight < 47 kg and high DMFT index scores (≥ 24) did not remained significantly different in a subsequent logistic regression analysis adjusted for age and sex. Independent variables were age, sex, and high DMFT index score (≥ 24). The dependent variable was dry weight < 47kg. DMFT scores: the numbers of total, decayed (DT), missing (MT), and filled (FT) teeth.

^a^Bold values indicate statistical significance at *p*< 0.05.
