# Supplementary material for: Possible link between dental diseases and arteriosclerosis in patients on hemodialysis
Source: PLoS One. 2019 Dec 13;14(12):e0225038. doi: 10.1371/journal.pone.0225038 (PMC6910673; doi:10.1371/journal.pone.0225038)
Supplement: S2 Table — (DOCX) [file pone.0225038.s002.docx]

**S2 Table: Association between BMI<20 and High DMFT Index Score in Patients on Hemodialysis.**

| **Variables** | **Odds ratio (95% confidence interval)** | ***P*-value^a^** |
| --- | --- | --- |
| Age | 1.030 (0.988–1.074) | 0.1674 |
| Sex (Male) | 0.430 (0.168–1.102) | 0.0786 |
| High DMFT  (DMFT index score ≥ 24) | 1.547 (0.533–4.487) | 0.4220 |

Additive multivariate logistic regression models adjusted for age and sex were used for these analyses in patients on HD. The association between BMI<20 and high DMFT index scores (≥ 24) did not remained significantly different in a subsequent logistic regression analysis adjusted for age and sex. Independent variables were age, sex, and high DMFT index score (≥ 24). The dependent variable was BMI<20. BMI: body mass index.DMFT scores: the numbers of total, decayed (DT), missing (MT), and filled (FT) teeth.

^a^Bold values indicate statistical significance at *p*< 0.05.
